# Possible Transmission of Pandemic (HIN1) 2009 Virus with Oseltamivir Resistance

**DOI:** 10.3201/eid1605.091835

**Published:** 2010-05

**Authors:** Michal Mandelboim, Musa Hindiyeh, Tal Meningher, Ella Mendelson

**Affiliations:** Chaim Sheba Medical Center, Ramat-Gan, Israel (M. Mandelboim, M. Hindiyeh, T. Meningher, E. Mendelson); Bar-Ilan University, Ramat-Gan (T. Meningher); Tel-Aviv University, Ramat Aviv, Israel (E. Mendelson)

**Keywords:** Pandemic (HIN1) 2009, oseltamivir resistance, influenza, viruses, expedited, letter

**To the Editor:** In March 2009, a new strain of influenza A (H1N1) virus of swine origin emerged; the virus had crossed the species barrier to humans and acquired the capability of human-to-human transmission. Soon after, the World Health Organization raised the worldwide pandemic alert to level 6 (www.who.int/en), declaring the first influenza pandemic in the past 42 years. The virus was named influenza A pandemic (H1N1) 2009 virus. The illness caused by this virus is particularly dangerous for pregnant women and for patients with chronic diseases (*1*). The preferred treatment is a neuraminidase inhibitor, zanamivir or oseltamivir (*2*).

Around the world, several dozen cases of resistance to oseltamivir in persons with or without exposure to the drug have been reported (*3*). However, only limited information is available with regard to initial infections with oseltamivir-resistant viruses (*4*). We report a case of possible human-to-human transmission of pandemic (H1N1) 2009 virus in Israel.

After the recent discovery of oseltamivir-resistant strains, we conducted a retrospective study of oseltamivir-resistance mutations in viral RNA amplified from specimens from patients hospitalized >1 week with pandemic (H1N1) 2009. All samples were first tested for the H275Y mutation by using an in-house real-time reverse transcription–PCR (RT-PCR) assay developed at the Central Virology Laboratory of Chaim Sheba Medical Center; positive results were confirmed by sequencing. The histidine-to-tyrosine mutation at the 275 position of the neuraminidase protein results in reduced binding of oseltamivir.

During June–August 2009, ≈80 children in an institution for disabled children were suspected of being infected with pandemic (H1N1) 2009 virus. The children had influenza-like signs and symptoms, and at that time the only influenza virus circulating in Israel was pandemic (H1N1) virus. Of these 80 patients, 10 were hospitalized because of the severity of their clinical signs or disease complications, and for 7, RNA of the pandemic (H1N1) 2009 virus was detected in throat and nasal swabs by real-time RT-PCR.

Patient 1 was a 13-year-old boy with cerebral palsy and partial blindness, who was treated with oseltamivir (60 mg twice a day) for 5 days (July 27–31, 2009). After some improvement, his condition worsened, and he was hospitalized on August 13 for breathing difficulty and high fever. Real-time RT-PCR indicated infection with pandemic (H1N1) 2009 virus. During our survey, we found that patient 1 was infected with a virus carrying the H275Y mutation, suggesting that the mutation evolved during oseltamivir treatment.

Patient 2 was a 10-year-old girl who had lived in the room next to patient 1 and who also had cerebral palsy. Her signs and symptoms started on August 13, 2009, and she was hospitalized on August 15. She was treated in the hospital for 5 consecutive days with oseltamivir, steroids, and augmentin; she was discharged on August 21. Her diagnosis was made early in the clinical course of her infection, and she was infected with pandemic (H1N1) 2009 virus carrying the H275Y mutation.

In contrast, none of the other 8 children who were hospitalized for pandemic (H1N1) 2009 carried the mutation. Although we cannot rule out the possibility that the virus was transmitted by a third person, we suggest that the virus carrying the resistance mutation was probably transmitted from patient 1 to patient 2. This transmission is probable because these 2 patients lived in adjacent rooms, they were in contact with each other, clinical onset of patient 1 preceded that of patient 2 by a few days, and patient 2 had the mutation at the beginning of her disease.

Fortunately, despite the conditions that favor virus spread in such institutions, this virus did not seem to spread further; the other 8 hospitalized children from this institution were infected with the wild-type virus. Nevertheless, the potential for spread of pandemic (H1N1) 2009 virus carrying the oseltamivir resistance mutation exists, thereby emphasizing the urgent need for a vaccination to prevent illness and for alternative drugs to treat infected patients.
